# Prevalence of and Risk Factors Associated with Alcohol Overconsumption at 2 Years After Bariatric Surgery

**DOI:** 10.1007/s11695-022-06060-6

**Published:** 2022-04-25

**Authors:** Lara Siikaluoma, Erik Stenberg, Mustafa Raoof

**Affiliations:** 1 School of Medical Sciences, Örebro University, Örebro, Sweden; 2 Department Surgery, Faculty of Medicine and Health, Örebro University, Örebro, Sweden

**Keywords:** Bariatric surgery, Weight loss surgery, Alcohol overconsumption, Alcohol use disorder, Phosphatidylethanol

## Abstract

**Introduction:**

Alcohol overconsumption remains one of the adverse effects associated with bariatric surgery. Many previous studies have used subjective methods to evaluate the prevalence of alcohol overconsumption. In 2018, Örebro University Hospital started to use phosphatidylethanol 16:0/18:1 (PEth) as a screening tool pre- and postbariatric surgery. Research exploring alcohol use after bariatric surgery assessed with PEth is scarce.

**Aim:**

The aim of this study is to evaluate the prevalence of alcohol overconsumption in bariatric surgery patients measured 2 years postoperatively with PEth and to identify possible risk factors associated with alcohol overconsumption.

**Methods:**

This was a register-based retrospective, observational cohort study with PEth results collected from medical records at Örebro University Hospital. Patients who underwent bariatric surgery between January 2016 and June 2019 and who were registered in the Scandinavian Obesity Surgery Registry (SOReg) were included.

**Results:**

PEth results from 410 bariatric surgery patients were identified. PEth values significantly increased from baseline to the postoperative follow-up (from 3.0% before surgery to 8.3% at the 2-year follow-up). In a univariate logistic regression analysis, the associated risk factors were found to be male sex (odds ratio, OR 2.14), older age (OR 1.06), and hypertension (OR 3.32).

**Conclusion:**

The prevalence of alcohol overconsumption measured with PEth 2 years after bariatric surgery was 8.3% and was associated with male sex, older age, and hypertension. More studies are needed to validate the results of this study because it is not known whether PEth values are affected by bariatric surgery.

**Graphical abstract:**

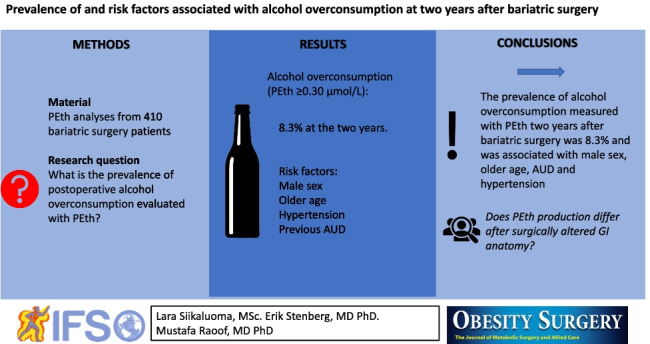

## Introduction

Significant changes in alcohol metabolism have been observed after Roux-en-Y gastric bypass (RYGB), such as a higher peak concentration of alcohol, faster absorption, and a longer time to become sober [1, 2]. Alterations in alcohol metabolism may result in an increased risk for alcohol use disorders (AUDs) after surgery, a risk that persists over time [3, 4]. However, it has been suggested that AUD after bariatric surgery may not represent new-onset AUD but rather a relapse after full sustained remission around the time of bariatric surgery [5]. Alternatively, there are theories suggesting that people with obesity have changed one addiction for another; in this case, it is alcohol to food, and after bariatric surgery, they change back from food to alcohol [5–7]. Phosphatidylethanol (PEth), an abnormal phospholipid, can be used as an alcohol marker to detect and monitor alcohol abuse; moreover, it can be used to identify early harmful alcohol use with a theoretical diagnostic specificity of 100% because PEth is formed only when alcohol is present [8]. PEth can be detected for up to 30 days, but the results can be affected by how often alcohol is consumed and the blood alcohol concentration [9]. There are different homologs of PEth, but the most common homolog is 16:0/18:1, representing 46% of total PEths [10, 11]. In a prospective multicenter cohort study evaluating the Alcohol Use Disorders Identification Test (AUDIT), the prevalence of AUD was 7.3% 1 year after bariatric surgery, increasing in prevalence to 9.6% 2 years after surgery [12]. A meta-analysis and systematic review by Azam et al. found that there was no significant difference in the prevalence of AUD 1 and 2 years postbariatric surgery, but the prevalence increased significantly 3 years after the RYGB procedure [13]. All of these studies were based on self-reported alcohol use. Studies evaluating alcohol use with PEth are limited. In a prospective cohort study, Walther et al. used a reference group of healthy blood donors and two separate groups of patients undergoing RYGB to assess alcohol intake with PEth at preoperative baseline and at 1 and 2 years postoperatively. The study found that the PEth levels of the bariatric surgery groups were lower than those of the reference group at baseline and postoperatively; notably, in both bariatric surgery groups, the PEth levels increased significantly postoperatively compared to baseline but did not reach the PEth levels of the control group. However, this study included only PEth ≥ 0.05 μmol/L without classifying it further as 0.05–0.30 μmol/L or > 0.30 μmol/L; in addition, the prevalence of alcohol overconsumption and risk factors were not examined [14]. The aim of this study was to evaluate the prevalence of alcohol overconsumption at 1 and 2 years after bariatric surgery and to identify possible risk factors associated with alcohol overconsumption.

## Material and Methods

### Study Design

This was a retrospective observational cohort study based on prospectively collected data registered in the Scandinavian Obesity Surgery Registry (SOReg). The SOReg, which has been shown to have high data quality and is constantly validated, covers 98% of all bariatric procedures performed in Sweden since 2009 with records entered in the database preoperatively, at surgery and at outpatient visits at 6 weeks and 1, 2, and 5 years after surgery [15]. Data from the SOReg includes information on patient demographics, postoperative complications, associated medical problems, and weight measurements. Associated medical problems were specified as specific conditions (diabetes, depression, dyslipidemia, hypertension, or sleep apnea) treated with pharmacological treatment or nocturnal continuous positive airway pressure in the case of sleep apnea. Previous AUD was defined as a previous diagnosis from a specialized unit or in the case of previous use of medication for alcohol disorder (Anatomical Therapeutic Chemical code: N07BB). PEth results were collected retrospectively from the electronic patient records at Örebro University Hospital and registered within the SOReg database. All available baseline and postoperative PEth results were included.

### Study Population

All patients who were eligible for and underwent sleeve gastrectomy (SG) or RYGB at Örebro University Hospital and a County Hospital between January 2016 and June 2019 with a registration in the SOReg were included in this study. To be eligible for bariatric surgery after fulfilling the BMI requirements (BMI ≥ 35 kg/m^2^ with or without associated medical problems), patients need to be free from drug or alcohol abuse for at least 2 years before surgery if they have any history of abuse [16]. Bariatric surgery was performed by the same team; the standard RYGB procedure followed the H. Lönroth method [17, 18], and the SG procedure followed the New York summit consensus [19].

### PEth Measurements

PEth was introduced as a routine measurement in this patient cohort in 2018. The results were registered within the clinical registry. Alcohol overconsumption was defined as PEth > 0.30 μmol/L; moderate alcohol consumption, PEth 0.05–0.30 μmol/L; and low or no alcohol consumption, PEth < 0.05 μmol/L, as suggested by Equalis, an expert group for pharmaceuticals and toxicology, to standardize the PEth results in Sweden [20]. PEth results were obtained from blood samples analyzed in accordance with regional laboratory guidelines.

### Statistical Methods

The prevalence of alcohol consumption and patients’ baseline demographics were analyzed using descriptive statistical methods. Differences between the prevalence of alcohol overconsumption (PEth > 0.30 μmol/L) and at least moderate consumption (PEth > 0.05 μmol/L) from baseline to 1 and 2 years were assessed for statistical significance with the χ2 test. Associated baseline factors for alcohol overconsumption were evaluated using an unadjusted logistic regression analysis, with odds ratios (ORs) and 95% confidence intervals (CIs) as measures of association. *P* values < 0.05 were considered indicative of statistical significance. All analyses were carried out using IBM SPSS Statistics MAC version 26.0 (Armonk, NY, USA).

## Results

### Collected Data

During the study period, 621 patients were included in the SOReg and followed for 2 years after surgery. Since PEth was introduced as a routine measurement in this patient cohort in 2018, samples were not available for all patients at all points in time. Of the 621 patients included, 236 (84.9%; of those with potential registration) had a registered PEth result at baseline, 381 (61.4%; of those with potential registration) had a result at 1 year, and 410 (66.0%; of those with potential registration) had a result at 2 years. In total, 114 patients (18.4%) had PEth results registered at all three time points (baseline and 1 and 2 years, postoperatively).

### Preoperative and Postoperative Characteristics of the Study Cohort

Table 1 shows the preoperative characteristics of the study cohort at baseline. The mean age was 40 years, 73% were females, and the mean preoperative BMI was 42. At 2 years after surgery, remission of several metabolic associated *medical problems* was seen, but more patients were current smokers; in addition, a higher prevalence of clinical depression was noted (Table 2).

**Table 1:** Preoperative (baseline) demographics and characteristics of the study cohort

Variable	Preoperative cohort, *n* = 621
Age (y), mean ± SD	40.2 ± 12.4
Preoperative BMI, mean ± SD	42.0 ± 5.5
Sex, *n* (%)	
Female	455 (73.3)
Male	166 (26.7)
Associated metabolic problems, *n* (%)	
Diabetes	80 (12.9)
Depression	52 (8.4)
Dyslipidemia	42 (6.8)
Hypertension	153 (24.6)
Sleep apnea	83 (13.4)
Previous AUD^1^, *n* (%)	29 (4.7%)
Current smoker, *n* (%)	85 (14.0%)

*BMI* body mass index. *SD* standard deviation. *AUD* alcohol use disorder

^1^Missing data for 1 patient (0.2%)

**Table 2:** Demographics and characteristics of the study cohort at 2 years postoperatively, divided into groups according to alcohol overconsumption (PEth > 0.30 μmol/L) or not

Variable	Entire cohort	Alcohol overconsumption	No alcohol overconsumption	*P* value
Age, mean ± SD	43.0 ± 12.4	50.8 ± 9.9	42.3 ± 12.3	< 0.001
Preoperative BMI, mean ± SD	41.7 ± 5.2	41.2 ± 5.3	41.8 ± 5.2	0.541
Change in BMI, mean ± SD	-13.6 ± 4.4	-12.9 ± 4.3	-13.6 ± 4.4	0.336
%EBMIL, mean ± SD	84.1 ± 24.1	84.2 ± 28.6	84.0 ± 23.6	0.981
Sex, *n* (%)				
Female	307 (74.9%)	20 (58.8%)	287 (76.3%)	Reference
Male	103 (25.1%)	14 (41.2%)	89 (23.7%)	0.027
Surgical method, *n* (%)				
Gastric bypass	289 (70.5%)	26 (76.5%)	263 (70.0%)	Reference
Sleeve gastrectomy	121 (29.5%)	8 (23.5%)	113 (30.0%)	0.426
Associated medical problems, *n* (%)				
Current smoker^2^	68 (20.2%)	7 (23.3%)	61 (19.9%)	0.659
Diabetes^3^	20 (5.4%)	5 (15.2%)	15 (4.4%)	0.015
Depression^3^	50 (13.4%)	5 (15.2%)	45 (13.3%)	0.763
Dyslipidemia^3^	27 (7.3%)	5 (15.2%)	22 (6.5%)	0.076
Hypertension^3^	67 (18.0%)	14 (42.4%)	53 (15.6%)	< 0.001
Sleep apnea^3^	13 (3.5%)	2 (6.1%)	11 (3.2%)	0.408

*BMI* body mass index. *SD* standard deviation; *EBMIL* excess BMI loss

^1^Alcohol overconsumption was defined as PEth > 0.30 μmol/L

^2^*P* values based on unadjusted logistic regression analysis

^3^Missing data from 38 patients. Depression defined as if the patient was receiving pharmacological treatment against depression

^4^Missing data from 74 patients

### Characteristics of the Study Cohort at the 2-Year Follow-Up

At baseline before bariatric surgery, the prevalence of alcohol overconsumption (PEth > 0.30 μmol) was 3.0%. The prevalence of alcohol overconsumption at the 1-year follow-up was 6.8% and at 2 years 8.3%. The number of patients with low or no alcohol consumption declined with time after surgery (Table 3).

**Table 3:** Prevalence of alcohol consumption levels among the 621 surgical patients who had PEth measurements taken at baseline, 1 year and/or 2 years

PEth (μmol/L)^1^	Baseline, *n* = *236*	1 year, *n* = *381*	*P*^*2*^ 1 year vs. baseline	2 years, *n* = *410*	*P*^*2*^* 2 years* vs. baseline
< 0.05, *n* (%)	193 (81.8)	285 (74.8)	Reference	303 (73.9)	Reference
0.05–0.30, *n* (%)	36 (15.3)	70 (18.4)		73 (17.8)	
> 0.30, *n* (%)	7 (3.0)^3^	26 (6.8)	**0.038**	34 (8.3)	**0.007**

*PEth* phosphatidylethanol

^1^PEth < 0.05 μmol/L was defined as low or no alcohol consumption; PEth 0.05–0.30 μmol/L, as moderate alcohol consumption; and PEth > 0.30 μmol/L, as alcohol overconsumption

^2^*P* values based on the χ2 test

^3^At that time period, we had not established reference values for the use of PEth values in the evaluation of this as a contraindication for bariatric surgery. Thus, 7 patients whom we would now consider to have contraindications for surgery were included (based on best available information at that time)

Patients with high alcohol overconsumption at 2 years were more likely to be older and male. They were more likely to require pharmacological treatment for diabetes and hypertension 2 years after surgery (Table 2). RYGB was the predominant surgical method in all groups, with no difference in alcohol overconsumption between the surgical methods. Increased age, male sex, and hypertension at baseline were all associated with a higher risk for AUD at 2 years after surgery (Table 4).

**Table 4:** Baseline factors associated with an increased risk for alcohol overconsumption (PEth > 0.30 μmol/L)

Variable	OR (95% CI)	*P* value^1^
Age, mean ± SD	1.06 (1.03–1.09)	< 0.001
Preoperative BMI, mean ± SD	0.98 (0.92–1.06)	0.644
Sex, *n* (%)^1^		
Female	Reference	Reference
Male	2.14 (1.05–4.39)	0.037
Surgical method, *n* (%)^1^		
Gastric bypass	Reference	Reference
Sleeve gastrectomy	0.69 (0.30–1.56)	0.369
Current smoker^2^	1.20 (0.44–3.24)	0.726
Associated medical problems		
Diabetes	2.07 (0.89–4.82)	0.092
Depression	NA	1.000
Dyslipidemia	2.15 (0.77–5.98)	0.144
Hypertension	3.32 (1.64–6.73)	0.001
Sleep apnea	1.74 (0.72–4.22)	0.408
Previous AUD	3.57 (1.10–11.54)	0.034

*BMI* body mass index. *SD* standard deviation; *EBMIL* excess BMI loss. *AUD* alcohol use disorder

^1^Based on unadjusted logistic regression analysis

^2^Missing data from 16 patients

## Discussion

Alcohol overconsumption increased over time, with a prevalence of alcohol overconsumption of 8.3% at 2 years postoperatively. Male sex, older age, hypertension, and previous AUD were associated with alcohol overconsumption 2 years after bariatric surgery.

Walter et al. reported a prevalence of alcohol consumption (PEth > 0.05 μmol) of 26% 2 years after surgery among RYGB patients, which is in agreement with the alcohol consumption in our study (26.1%). In their study, a group of healthy blood donors was used as the reference, with PEth results > 0.05 μmol found in 44% of the controls, indicating that there could be higher alcohol consumption in the general population than among bariatric surgery patients [14].

Many previous studies have used the Alcohol Use Disorders Identification Test (AUDIT/AUDIT-C) questionnaires [3, 4, 12, 21], reporting an increased prevalence of AUD from the first to second postoperative year, reaching 9.6% at 2 years after surgery [12]. While comparisons between studies using self-estimates and PEth values may not be directly comparable, PEth results have been shown to overlap with AUDIT-C results [22–24]. The rate of AUD in our study reflects those reported in previous studies that have defined alcohol overconsumption with more subjective methods.

In our study, the risk of AUD increased within the first postoperative year and increased further over time. Similar findings have been reported previously in studies using AUDIT-based definitions as well [4, 12]. Interestingly, one study showed that the prevalence of new-onset alcohol overconsumption was 7% 1 year after bariatric surgery and 6% after 2 years, whereas more than 50% of baseline high-risk drinking resolved after surgery [21]. In the context of our study, this could indicate that many of the patients with AUD at follow-up had developed new-onset alcohol overconsumption after surgery.

Another possible explanation for the increase in alcohol overconsumption after bariatric surgery could be a relapse in alcohol use rather than the development of a new-onset alcohol problem. Patients with a lifetime history of AUD who undergo bariatric surgery are more likely to develop an AUD postoperatively, although patients who underwent bariatric surgery in the USA have been reported to have a lifetime prevalence of AUD comparable to that of the general population [5]. In line with our findings, male sex has been documented as a risk factor for postsurgery AUD [3, 12]. Moreover, previous studies have reported that smoking and younger age are associated with an increased risk for AUD after bariatric surgery [3, 12]. In contrast, in our study, smoking did not influence the risk of alcohol overconsumption 2 years postbariatric surgery. Furthermore, older age was associated with a higher risk for overconsumption.

An unexpected finding in our study was the lack of any significant association between depression and postoperative alcohol overconsumption. In fact, none of the 56 patients who were treated for clinical depression at baseline developed AUD. This finding is supported by King et al., who also investigated preoperative mental health, binge eating, and psychiatric treatment [12].

Last, we did not see any difference in alcohol overconsumption between the RYGB and SG groups, which is also in agreement with another study and in accordance with the fact that SG also significantly alters alcohol metabolism [25, 26].

Patients who developed AUD more often had metabolic *associated medical problems* (in particular in the form of hypertension) at baseline. While it remains unlikely that hypertension per se should be associated with the development of AUD, patients with hypertension tend to be older and may also share additional factors associated with both hypertension and AUD. In addition, patients who did develop AUD had higher rates of metabolic associated *medical problems* (including hypertension) at 2 years after surgery. This finding is consistent with previous reports of a linear increase in the risk of hypertension when alcohol consumption increases [27]. While bariatric surgery improves hypertension, diabetes, and other metabolic *associated medical problems*, alcohol overconsumption has been reported to increase the risk of hypertension threefold [28], which is in line with the findings of the present study.

Despite the strengths of a well-defined cohort, the inclusion of RYGB and SG, and the administration of standardized counseling, follow-up, and supplementation, there are potential limitations that may affect the interpretation of the results in this study. First, because of the retrospective nature of this study, there were missing data. Since screening with PEth started in 2018 and our study cohort included patients who underwent surgery before that, there were missing data, especially at baseline and 2 years after surgery, affecting the reliability of the results. One could expect that there was no selection bias because patients with the same eligibility criteria underwent surgery before and after the beginning of PEth screening. During the study period, the evaluation of contraindications for bariatric surgery relied on self-reported alcohol use and review of hospital charts which explains why seven patients with a PEth value ≥ 0.30 still underwent surgery.

In addition, this is a small patient sample, which affects the generalizability of the results. Finally, to our knowledge, whether the production of PEth in response to alcohol intake differs when gastrointestinal anatomy is surgically altered remains unknown.

## Conclusion

The prevalence of alcohol overconsumption measured with PEth 2 years after bariatric surgery was 8.3% and was associated with male sex, older age, AUD, and hypertension.
